# Bradycardia, Renal Failure, Atrioventricular Nodal Blockade, Shock, and Hyperkalemia (BRASH) Syndrome as a Presentation of Coronavirus Disease 2019

**DOI:** 10.7759/cureus.7816

**Published:** 2020-04-24

**Authors:** Vishaal Prabhu, Edmund Hsu, Stephan Lestin, Yasamin Soltanianzadeh, Sara Hadi

**Affiliations:** 1 Emergency Medicine, Mount Sinai St. Luke's - Roosevelt Hospital Center, New York City, USA; 2 School of Medicine, University of Rochester School of Medicine and Dentistry, Rochester, USA

**Keywords:** coronavirus disease 2019, brash syndrome, sars-cov-2 (severe acute respiratory syndrome coronavirus -2), bradycardia, renal failure, av nodal blockers, shock, hyperkalemia

## Abstract

The novel coronavirus disease 2019 (COVID-19) has led to a global pandemic. While acute respiratory failure has been the predominant concern, there have been reports of other end-organ damage such as renal failure. We report a case of an elderly woman who presented with BRASH syndrome, a constellation of bradycardia, renal failure, atrioventricular (AV) nodal blockade, shock, and hyperkalemia (BRASH), which was likely triggered by COVID-19.

## Introduction

The novel coronavirus disease 2019 (COVID-19) caused by severe acute respiratory syndrome coronavirus 2 (SARS-CoV-2) has led to a pandemic. In the United States alone, at least 554,849 cases and 21,942 total deaths have been reported as of April 13, 2020 [1]. The common clinical manifestations of COVID-19 include fever, cough, fatigue, and shortness of breath, with acute respiratory distress syndrome (ARDS) being the most prominent complication. Here, we report a case of bradycardia, renal failure, AV nodal blockade, shock, and hyperkalemia (BRASH) syndrome as the initial presentation of COVID-19. This case report highlights the importance of recognizing and appropriately managing the uncommon clinical presentations of COVID-19.

BRASH syndrome is most often seen in elderly patients on long-term management with beta-blockers (BB) or calcium channel blockers (CCB) for cardiac disease. Triggers include hypovolemia, up-titration of home medications such as potassium-sparing diuretics or anti-hypertensives, sepsis, and other causes of acute kidney injury [2]. Patients with BRASH syndrome develop renal failure that leads to hyperkalemia. This has a synergistic effect with AV node blockers, i.e., BB and CCB, with the subsequent development of profound hypotension and bradycardia [3-4]. A key characteristic of BRASH syndrome is mild hyperkalemia causing significant bradycardia without other, typical electrocardiogram (EKG) changes (i.e., widening QRS, peaked T-waves). Bradycardia leads to hypotension, poor renal perfusion, and worsening of renal failure, resulting in a vicious cycle. Standard advanced cardiac life support (ACLS) algorithms may fail in reversing the bradycardia seen in these patients so it is important to recognize this syndrome and address the underlying etiology in addition to the synergistic effects of AV nodal blockade and hyperkalemia.

## Case presentation

An elderly woman presented to a New York City urban academic emergency department by ambulance for syncope, hypotension, and bradycardia. Her blood pressure (BP) in the field was 80/45 mmHg and heart rate (HR) was 33 beats per minute (bpm). Prior to arrival, the patient had a right tibial intraosseous (IO) line placed, was given 0.5 mg atropine twice, and was started on a 2 mcg/kg/min dopamine drip. Her fingerstick glucose (FSG) pre-hospital was 200 mg/dL. Past medical history was notable for chronic kidney disease and hypertension, for which the patient was taking aspirin, carvedilol, and verapamil.

Initial vital signs in the emergency department were a temperature of 36.3 degrees Celsius, an HR of 45 bpm, a BP of 80/50 mmHg, a respiratory rate of 20, and oxygen saturation of 94% on nasal cannula at 6 liters per minute. On physical exam, her airway was patent and she had clear bilateral breath sounds. Her distal pulses were intact but difficult to palpate and her extremities were cold to the touch. The patient was arousable to voice and painful stimuli and was oriented to name.

**Figure 1:**
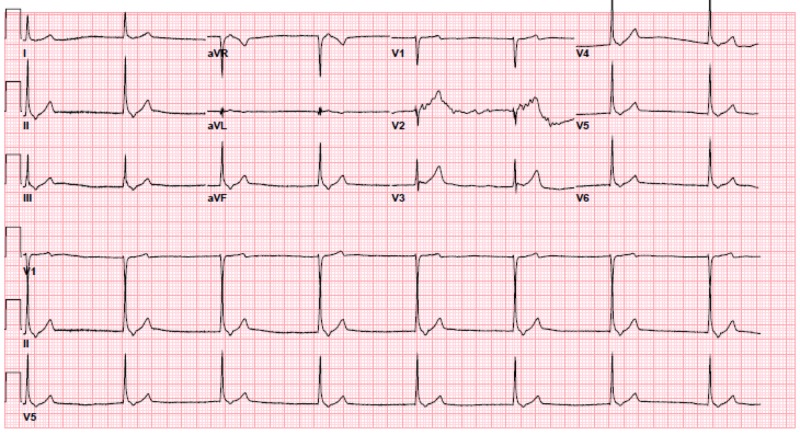
Initial EKG - junctional rhythm at 45 bpm EKG: electrocardiogram

Upon arrival, pacer pads were immediately placed. Peripheral access was difficult to obtain and the pre-hospital IO line did not flush well, so a crash left femoral central venous catheter was inserted. Her electrocardiogram (EKG) showed a junctional rhythm at a rate of 45 bpm without ischemic changes (Figure 1). A prior EKG from 18 months ago showed normal sinus rhythm (NSR) at a rate of 85 bpm. The point of care ultrasound showed no cardiac tamponade and good cardiac contractility.

The patient was given another 0.5 mg of atropine. Her potassium on a point of care venous blood gas was 6.8 mEq/L, so 1 gram of calcium gluconate, 5 units of regular insulin, 1 ampule of sodium bicarbonate, and 1 L of normal saline were administered. Her basic metabolic panel (BMP) was notable for a potassium of 6.5 mEq/L, a creatinine of 3.01 mg/dL (previously 1.84 - 2.04 mg/dL), a blood urea nitrogen (BUN) of 64 mmol/L, and a glucose of 283 mg/dL.

**Figure 2:**
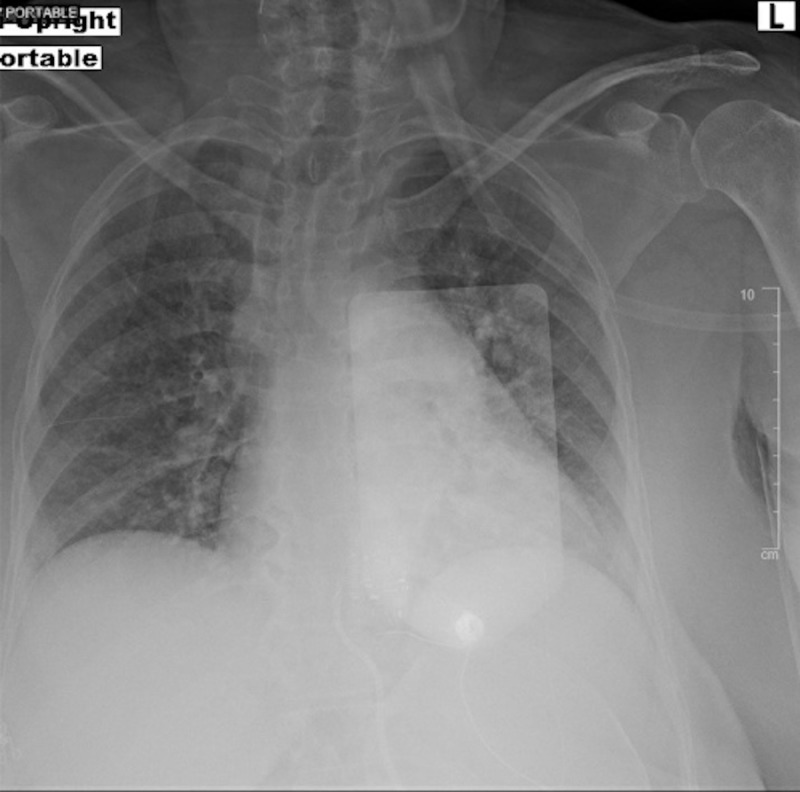
Portable chest X-ray on presentation showing diffuse, hazy, ground-glass airspace disease

**Figure 3:**
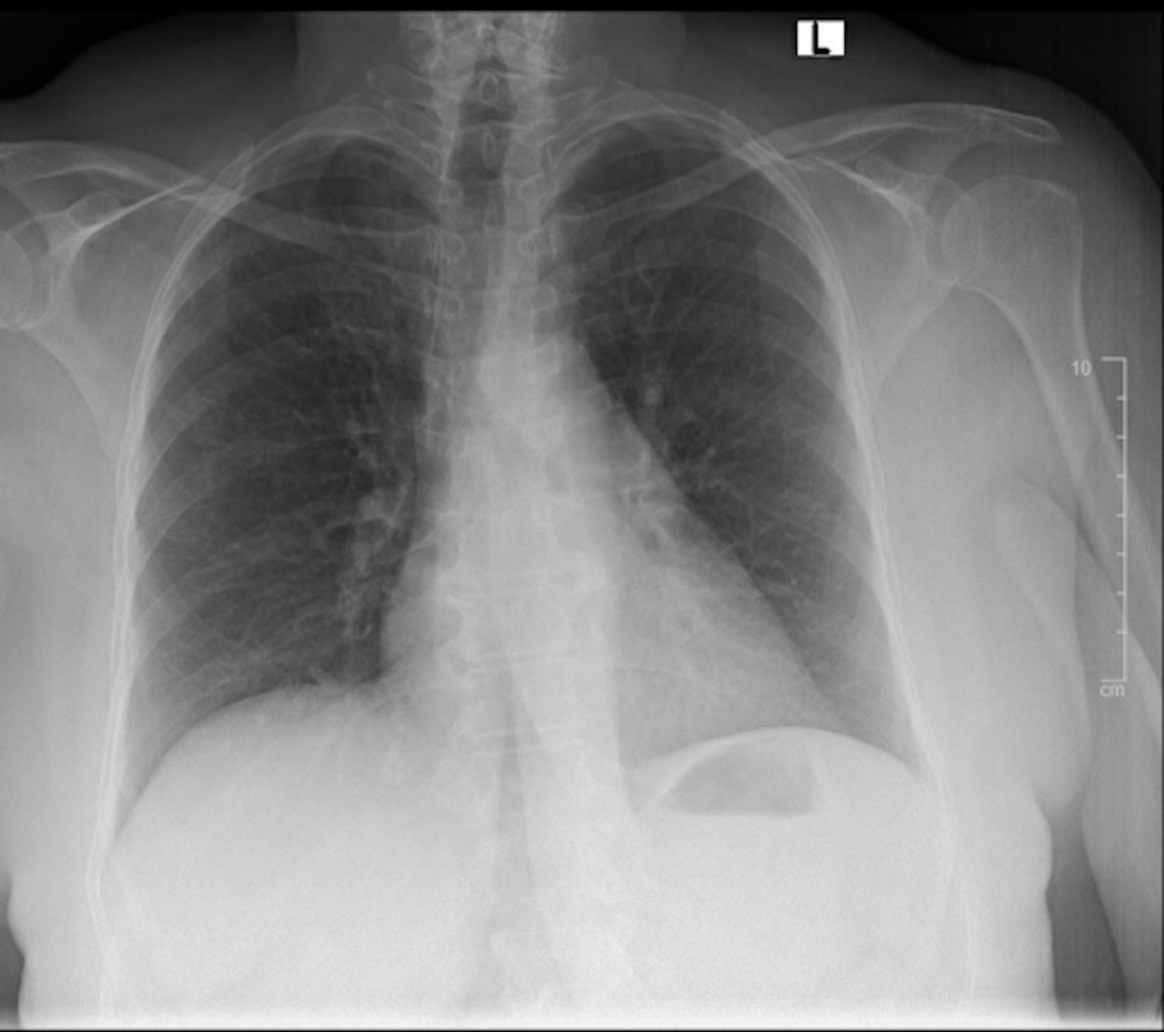
Previous chest X-ray from 2018 showing no acute pulmonary disease

**Figure 4:**
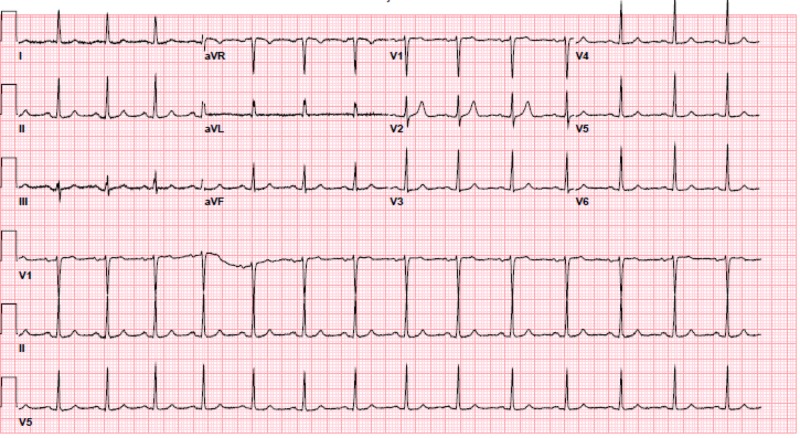
Repeat EKG - normal sinus rhythm at 87 bpm EKG: electrocardiogram

The patient’s hypotension, bradycardia, and hyperglycemia raised suspicion for calcium channel blocker toxicity as well as BRASH syndrome. The local poison control center, nephrology, and cardiology were consulted. The patient was given three additional grams of calcium gluconate, Zofran 4 mg IV, and glucagon 5 mg intravenous (IV). She was then started on a 10% dextrose drip at a rate of 100 mL/hr, given a 1U/kg bolus of insulin, and an insulin infusion at 1 U/kg/hr. A point of care fasting blood sugar (FSG) was checked every 15 minutes and a BMP was drawn every 30 minutes. A portable chest X-ray showed bilateral reticular opacities as well as mild hazy opacities in the bibasilar lungs, which were new compared to a prior chest X-ray taken 18 months prior (Figures 2-3). After one hour, the patient's BP improved from 80/45 mmHg to 100-120/60-80 mmHg and her HR improved from 45 bpm to 70-90 bpm. A repeat EKG showed normal sinus rhythm at 87 bpm (Figure 4). The patient had improvement in her mental status and reported she was feeling better. She remained stable and was admitted to the medicine floor.

Hospital course

The patient’s home anti-hypertensive medications, carvedilol and verapamil, were held and she remained stable in normal sinus rhythm on telemetry. She was confirmed to be positive for COVID-19 through SARS-CoV-2 PCR testing. The patient was hospitalized for seven days, during which she completed a five-day course of azithromycin and hydroxychloroquine. She was weaned off supplemental oxygen and her creatinine improved to 2.41 mg/dL. A formal transthoracic echocardiogram was unremarkable. The patient was discharged home with verapamil discontinued and her BB switched to metoprolol succinate. Close follow-up was arranged with her primary care physician and nephrologist.

## Discussion

The novel coronavirus disease 2019 (COVID-19) is caused by severe acute respiratory syndrome coronavirus 2 (SARS-CoV-2), a positive-sense, single-stranded ribonucleic acid (RNA) virus. The virus primarily enters cells by binding to angiotensin-converting enzyme-2 (ACE2) receptors, which are found in cells of many organs including the lungs, kidneys, intestines, and heart [5]. Person-to-person spread appears to occur primarily through respiratory droplets. The most common presenting symptoms include cough, fever, fatigue, shortness of breath, sore throat, and headache [6].

While acute respiratory failure is the primary complication seen in COVID-19, patients are at high risk for multiorgan failure, including renal failure. The true prevalence of acute kidney injury (AKI) in COVID-19 is still under investigation. One retrospective cohort study, which included 191 adult inpatients with laboratory-confirmed COVID-19 and definite clinical outcome (death or discharge) from Jinyintan Hospital and Wuhan Pulmonary Hospital, reported 15% of all patients and 50% of non-survivors had AKI [7]. In a cohort of 1,099 patients with laboratory-confirmed COVID-19 from 552 hospitals in China, only six (0.5%) had AKI [6]. A smaller study of 21 critically ill patients with confirmed COVID-19 infection admitted to an intensive care unit (ICU) in Washington state reported 19% had AKI [8]. While the overall prevalence of AKI in COVID-19 may be low, it is seen more frequently in the critically ill and may be associated with a higher risk for in-hospital death [9].

The exact cause of AKI in patients with COVID-19 is unclear and likely multifactorial. Given AKI often accompanies shock, acute tubular necrosis resulting from hypoperfusion is likely a common etiology. Other proposed mechanisms include cytokine storm syndrome resulting from sepsis and direct cellular injury through the virus binding to ACE2 receptors expressed on renal tubular cells [10].

To our knowledge, this is the first case report of a patient with COVID-19 presenting with BRASH syndrome, a vicious cycle of bradycardia, renal failure, AV nodal blockade, shock, and hyperkalemia. While the clinical entity has been previously described in the literature, the mnemonic “BRASH” was recently coined in a 2016 EMCrit article by Josh Farkas and remains an underrecognized syndrome [2].

The syndrome is a cascade of events, with the key being the synergism between hyperkalemia and AV nodal blockers that leads to reduced chronotropy and hypotension. The resulting hypoperfusion leads to worsening renal failure, which perpetuates the hyperkalemia and causes renally cleared AV nodal blockers to accumulate. BRASH syndrome has multiple potential triggers, including hypovolemia, sepsis, up-titration of home AV nodal blocking medications, and any cause of acute renal dysfunction. It contains characteristic effects of both hyperkalemia and AV nodal blocker toxicity. Studies have shown that verapamil, a CCB, in the presence of mild hyperkalemia, can accentuate AV conduction delay, leading to junctional bradycardia [3-4]. Thus, significant symptomatic bradycardia can be seen without the typical EKG changes seen in severe hyperkalemia such as a wide QRS or peaked T waves [2].

Traditional management of bradycardia following the ACLS algorithm, including the administration of atropine and cardiac pacing, may not be successful in patients with BRASH syndrome. Instead, treatment centers around therapy for hyperkalemia, addressing the underlying cause of renal injury (i.e., fluid resuscitation for hypovolemia) and sometimes treating BB or CCB toxicity. In managing hyperkalemia, IV calcium is utilized to stabilize the cardiac membrane while IV insulin and dextrose along with albuterol are used to shift potassium intracellularly. Albuterol may also assist in improving bradycardia. Bicarbonate can be considered in patients with acidosis. Ultimately, potassium must be excreted for definitive management. If patients are euvolemic and able to produce urine, potassium-wasting diuretics can be utilized with emergent dialysis considered in those refractory to treatment. Fluid resuscitation will be required in patients who are hypovolemic. For patients who remain hemodynamically unstable, the use of catecholamines such as dopamine or epinephrine may increase renal perfusion and help resolve the renal failure. Finally, treatment of AV nodal toxicity with high dose insulin and dextrose drip may occasionally be required [2,11-12].

Special precautions should be taken in patients with COVID-19, who appear to benefit from conservative fluid resuscitation [13]. However, those with BRASH syndrome who are hypovolemic require judicious fluids to improve renal function. Thus, volume status should be carefully assessed using tools like point of care ultrasound when deciding on fluid resuscitation in these patients. This is a delicate balance that requires special caution for ARDS and pulmonary tolerance of fluids.

## Conclusions

While pneumonia and ARDS are the primary complications seen in COVID-19, the disease has a wide range of clinical manifestations, including kidney dysfunction. In patients taking AV nodal blocking medications, renal failure can present as part of BRASH syndrome, a constellation of bradycardia, renal failure, AV nodal blockade, shock, and hyperkalemia. Clinicians taking care of patients during this global pandemic should be aware of uncommon presentations of COVID-19, such as BRASH syndrome, which may not respond to standard treatment algorithms.
